# Antibody Prevalence and Factors Associated with Exposure to *Orientia tsutsugamushi* in Different Aboriginal Subgroups in West Malaysia

**DOI:** 10.1371/journal.pntd.0002341

**Published:** 2013-08-01

**Authors:** Sun Tee Tay, Hafizatul Anis Mohamed Zan, Yvonne A. L. Lim, Romano Ngui

**Affiliations:** 1 Tropical Infectious Diseases Research and Education Centre (TIDREC), Department of Medical Microbiology, Faculty of Medicine, University of Malaya, Kuala Lumpur, Malaysia; 2 Department of Parasitology, Faculty of Medicine, University of Malaya, Kuala Lumpur, Malaysia; University of Texas Medical Branch, United States of America

## Abstract

**Background:**

Limited data is available on the current status of scrub typhus infection in the aboriginal population in Malaysia. This study was aimed to provide recent data on the degree of exposure of 280 individuals from seven aboriginal subgroups to *Orientia tsutsugamushi* (causative agent of scrub typhus) in West Malaysia. The environment, socioeconomic and behavioural risk factors associated with the disease were also investigated.

**Methods/Findings:**

The antibody prevalence to *O. tsutsugamushi* ranged from 0 to 36.4% in seven subgroups, with high prevalence rates noted in subgroups involved in agricultural activity and the lowest prevalence rates noted in subgroups whose main occupations were associated to fishing. Univariate analysis indicated populations with age above 18 years (OR = 1.15, 95% CI = 1.02–1.30, *P* = 0.015), working (OR = 1.99, 95% CI = 1.01–3.92, *P* = 0.044), working at agriculture area (OR = 1.18, 95% CI = 0.98–1.42, *P* = 0.031), receiving household income less than US$ 166.7 (RM500) per month (OR = 2.43, 95% CI = 1.16–5.11, *P* = 0.016) and having close contact with animal pets (OR = 4.06, 95% CI = 1.20–13.76, *P* = 0.016) are significantly associated with exposure to *O. tsutsugamushi*. Multivariate analysis confirms that participants who are above 18 years old, receiving household income less than US$ 166.7 (RM500) per month and having close contact with animal pets are 3.6 times (95% CI = 1.81–7.03, *P*<0.001), 1.3 times (95% CI = 1.14–1.64, *P* = 0.002) and 1.2 times (95% CI = 1.05–1.06, *P* = 0.006) more likely to have exposure to *O. tsutsugamushi*, respectively.

**Conclusion:**

The present study indicates that scrub typhus is still an important disease in the aboriginal population in Malaysia. Awareness about the disease and education on the preventive measures are important in reducing the risk of acquiring scrub typhus in the population studied.

## Author Summary

Scrub typhus has been recognized as a public health problem in the rural areas of Southeast Asia. The aboriginal population, one of the most impoverished groups in the Malaysia, may be exposed to the bite of *Leptotrombidium* mites (vector for scrub typhus) due to their involvement in the agricultural activities, living environment and personal habits. A serosurvey was conducted for seven aboriginal subgroups to determine antibody prevalence to *O. tsutsugamushi*, the causative agent of scrub typhus and to identify factors associated with scrub typhus. The findings in this study confirmed high antibody prevalence to *O. tsutsugamushi* in aboriginal subgroups who engaged in agricultural activity. Multivariate analysis showed that individuals who are above 18 years old, receiving household income less than US$ 166.7 (RM500) per month and having close contact with animal pets have higher exposure rates to scrub typhus. Institution of appropriate preventive measures is important in reducing the risk of acquiring scrub typhus in the population studied.

## Introduction

Scrub typhus is an acute febrile disease caused by *Orientia tsutsugamushi*, a Gram-negative obligate intracellular bacterium which is transmitted through the bites of infected *Leptotrombidium* mites. The disease is distributed throughout the Asia Pacific regions including Malaysia [1]–[3]. Also known as tsutsugamushi disease, the disease is characterized by focal or disseminated vasculitis and perivasculitis, which may involve the lungs, heart, liver, spleen, and central nervous system and cause serious complications including pneumonia, myocarditis, meningoencephalitis, acute renal failure, and gastrointestinal bleeding [3]–[5]. The disease has been reported as the most frequent infection among febrile hospitalized patients in rural areas of Malaysia since early 1970s [6]–[8], with antibody prevalence to *O. tsutsugamushi* varied widely from as low as 0.8% in East Malaysia [9] to as high as 73% in West Malaysia [6]. A recent serosurvey of febrile patients in rural areas of Malaysia showed a prevalence of 24.9% to *O. tsutsugamushi* [2].

According to Khor and Zalilah [10], the aborigines or orang Asli (translated as “original peoples”) are the indigenous inhabitants of West Malaysia, who constitute a minority group comprises only 0.6% of the total population of Malaysia. A total of 132,486 individuals have been recorded in a recent census [11]. They are officially classified into three main ethno-linguistic groups namely, the Senoi, Proto Malays or Aboriginal Malays and the Negritos, each consisting of six dialectic subgroups. The common occupations of the people are agricultural, fishery, hunting and collecting forest produce. Certain aboriginal subgroups such as Orang Laut, Orang Seletar and Mah Meri live close to the coast and are mainly fishermen. The Temuan, Jakun and Semai people are involved in agricultural activities for instance, in rubber, oil palm or cocoa plantations. The Temiar and Semelai live within forested areas and are engaged in rice cultivation, hunting and gathering. A minority of aboriginal population live in urban areas and are engaged in both waged and salaried jobs [12]. The aboriginal population has been identified as one of the most impoverished groups in the Malaysia, based on reports of various five-year Malaysia development plans [10].

Due to the life style and involvement in agricultural activities, high prevalence of scrub typhus has been reported from aboriginal populations in different geographical regions in Malaysia. Cadigan et al. [6] reported a prevalence of 73% in adult aborigines from “deep jungle”, 48% from “fringe areas”, and 8% from kampong (traditional villages). The incidence of scrub typhus infection varied from 3.2 to 3.9% per month in two aboriginal settlements in West Malaysia [13]. Molecular evidence of scrub typhus infections in the patients attending Hospital Gombak, a healthcare facility dedicated specifically for aboriginal population has also been reported [14]. However, little data is available on the assessment of the environment, socioeconomic and behavioural risk factors of the disease in different aboriginal subgroups in Malaysia.

This study was conducted to provide recent data on antibody prevalence and factors associated with exposure to *O. tsutsugamushi* infection in different aboriginal subgroups in West Malaysia. The information collected will be important for the improvement on management, prevention and control of scrub typhus in the aboriginal populations in Malaysia.

## Materials and Methods

### Ethical considerations

An ethical approval was obtained (i.e., MEC Ref. No. 824.11) from the Ethics Committee of the University Malaya Medical Centre (UMMC), Malaysia before the commencement of the study. The consent procedures regarding incompetent adults and the oral consent procedures had been approved by the ethical committee. An oral briefing on the objective and methodology of the study was given to the participants. Once they have voluntarily agreed to participate, their consents were taken either in written form (signed) or verbally followed by thumb prints (for those who were illiterate) of participants. Parents or guardians gave consent on behalf of all children. For incompetent adults, the questionnaires were completed by the head of the family who signed the informed consent on their behalf. All medical data was anonymized.

### Study population

**Figure 1:**
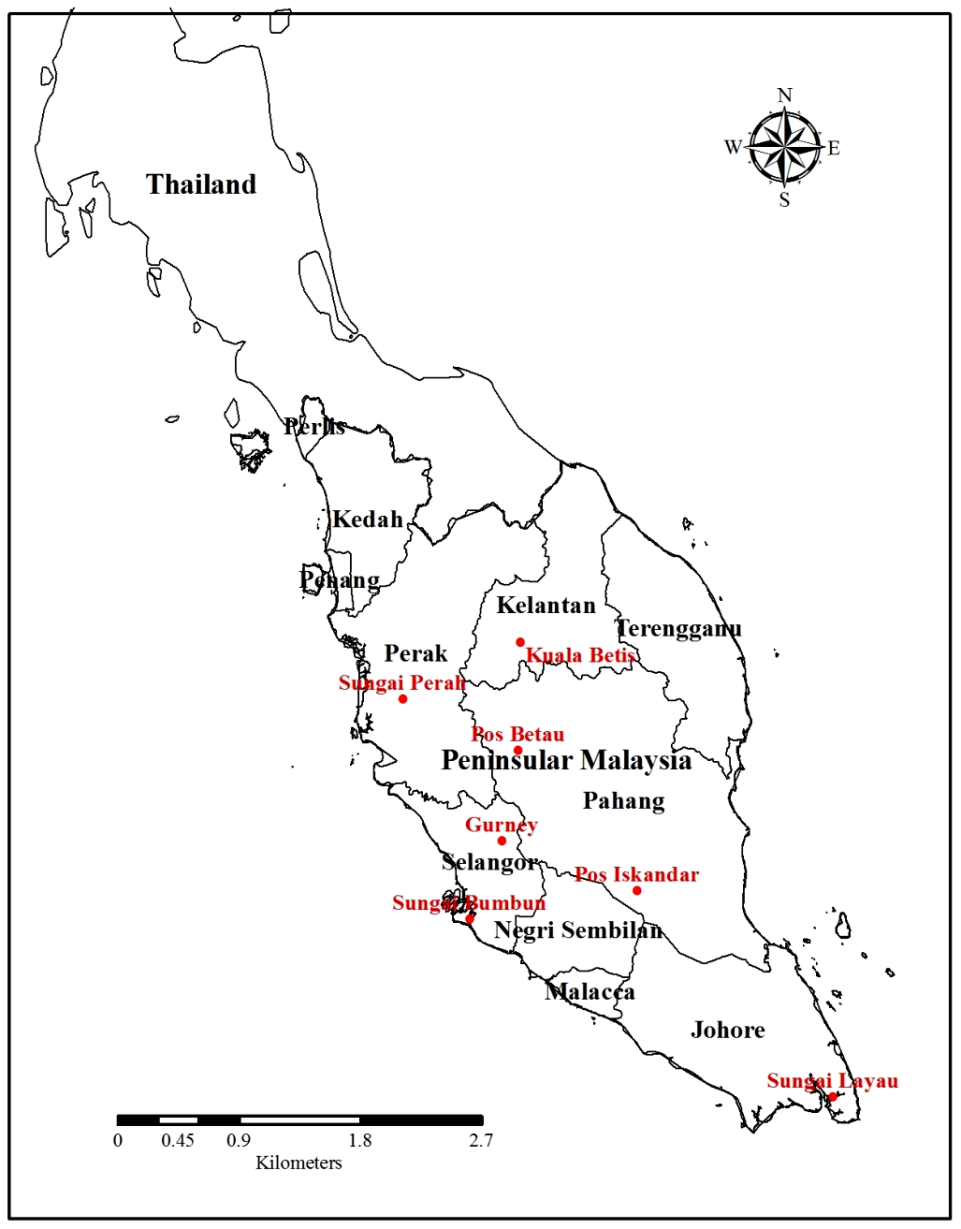
Location of the study areas in West Malaysia.

This study was a part of a large study to determine the occurrence and distribution of tropical infectious diseases among the aborigine populations. As there is no prior information about social and behavioural factors affecting scrub typhus for the aborigines, randomly selected serum samples from 280 individuals (representing approximately one third of the surveyed population) who participated in a serosurvey for prevalence and risk factors of intestinal parasitism in rural and remote West Malaysia from November 2007 to October 2010 were used in this study. At least 30 samples were selected from each study site, except for one study site (Sungai Bumbun) where only 14 samples were available for testing. Details of the consent, sample collection, sampling scheme and population prior to this study have been described previously [15]. The participants originated from 7 subgroups living in various states in West Malaysia, i.e., Temuan (Gurney; 101.44°E, 3.43°N), Semai Perak (Sungai Perah; 100.92°E, 4.48°N), Semai Pahang (Pos Betau; 101.78°E, 4.10°N), Semelai (Pos Iskandar; 102.65°E, 3.06°N) Temiar (Kuala Betis; 101.79°E, 4.90°N), Mah Meri (Sungai Bumbun; 101.42°E, 2.85°N) and Orang Kuala (Sungai Layau; 101.42°E, 2.85°N) (Figure 1). Of the seven subgroups selected in this study, five subgroups (i.e., Semelai, Semai Pahang, Temiar, Temuan and Semai Perak) are actively engaged in the agricultural activities whereas the remaining two (i.e., Orang Kuala and Mah Meri) live close to the coast and are involved in the fishing activities.

To determine the associated risk factors for scrub typhus infection, basic demographic data such as age, gender and education, socioeconomic status (i.e., occupation and household income) and behavioural aspects (i.e., personal hygiene such as wearing shoes, taking bath, and changing cloth and food consumption) of the participants were gathered from a questionnaire survey.

### Serologic analysis

The sera were analysed for IgG antibody against *O. tsutsugamushi* using a commercial assay (Scrub Typhus Detect IgG ELISA System, INBIOS International, Inc. USA) as in accordance to the manufacturer's instructions. The recombinant protein antigen (56-kda major outer membrane protein of the Karp strain of *O. tsutsugamushi*) used in the assay, has been reported to exhibit sensitivities and specificities similar to those of rickettsia-derived antigens in the indirect immunoperoxidase test, when evaluated using sera of individuals from Thailand, a neighbouring country at the northern part of Malaysia [16]. We believed that the assay is relevant and appropriate for screening of Malaysian population due to the many common factors shared by the two Southeast Asian countries, such as tropical climate and environment, types of chigger vectors and antigenic group of *O. tsutsugamushi*. Eight different serotypes of *O. tsutsugamushi* including the Karp strain have been identified in mites in Malaysia [17]. The distribution of Karp-related strains has also been found throughout various geographic regions in Southeast Asia [18].

Briefly, test serum and control were diluted 1∶ 100 using sample dilution buffer. The diluted serum samples were then transferred to the microtiter plate provided in the kit and incubated at 37°C for 30 min in a humidified incubator. The microtiter wells were washed six times and incubated for an additional 30 min at 37°C following the addition of secondary antibody (anti-human IgG conjugated with horseperoxidase). After incubation, the wells were washed six times before the addition of Enwash (a reagent provided by the manufacturer) followed by incubation at room temperature for 5 minutes. TMB substrate was then added to the microtiter wells and incubated for 10 min at room temperature in the dark. The reaction was stopped by adding Stop solution and the plate was read at 450 nm with a reference filter of 620 nm.

For determination of the cut-off value, the OD readings from the sera of 20 Malaysian blood donors (representing normal human serum) were obtained, averaged and added with three times of the standard deviation, as recommended by the manufacturer. A reading less than the cut-off value indicates a negative sample while a reading of more or equal to cut-off value is considered a positive sample.

### Statistical analysis

Statistical analysis was carried out using the SPSS (Statistical Package for the Social Sciences) software programme for Windows, version 17 (SPSS Inc., Chicago, IL). Before each analysis, initial data entry was cross-checked regularly (by HAMZ and RN) in order to be sure that data was entered correctly and consistently. The data with quantitative variables was expressed as means (± SD) and ranges, whereas, qualitative variables were estimated and presented as frequencies and percentages. A Pearson's Chi-square (χ^2^) test on proportion was used to test associations between variables. A univariate statistical model was used to assess potential associations between individuals with positive scrub typhus serological findings and the potential risk factors. In order to make sure that the potentially important predictors are not excluded and also due to a low number of predictor variables, all variables with or without lower significance level between 0.10 to 0.25 were included in the multivariate analysis using both backward and forward stepwise selection to produce the subset for final model, sequentially to determine significant differences in demographics and confounding risk factors among studied participants. The level of statistical significance was set up at p<0.05 and for each statistically significant factor, an odds ratio (OR) and 95% confidence interval (CI) were used for both univariate and multivariate logistic regression analysis to explore the strength of the association between scrub typhus seropositivity and the variable of interest.

## Results

### Demographic and baseline characteristics of the population

Table 1 shows the demographic and baseline characteristics of the 280 individuals surveyed in this study. The age of the participants ranged from 3 to 82 years (mean age = 22.6±16.3 years old). Majority of the participants were female (n = 168, 60.0%). The participants were divided into three age groups: those below 12 (15.7%), 12–17 (43.6%) and above 18 years old (40.7%). Of those below 18 years old, majority of them were students (n = 142, 50.7%). Of those above 18 years old, a minority of them (3.6%) were employed as labourers in factory while the remaining were rubber tappers (8.2%) and farmer/jungle produce gatherers (9.3%). A total of 28.2% of the participants were unemployed.

**Table 1:** Demographic and baseline characteristics of the surveyed population (n = 280).

Variable	no	(%)
**Age**		
Range	3–82 years	
Mean (± SD)	22.6 (±16.3)	
**Age groups (years)**		
Below 12	44	15.7
12 to 17	122	43.6
18 and above	114	40.7
**Gender**		
Female	168	60.0
Male	112	40.0
**Subgroups**		
Semelai	41	14.6
Semai Pahang	33	11.8
Temiar	73	26.1
Temuan	38	13.6
Semai Perak	33	11.8
Orang Kuala	48	17.1
Mah Meri	14	5.0
**Level of education**		
Formal education (at least 6-year of primary education)	160	57.1
No formal education	120	42.9
**Occupation categories**		
Not working	220	78.6
Working	60	21.4
**Occupation**		
Not working/housewife	79	28.2
Farmer/Jungle produce gatherer	26	9.3
Rubber tapper	24	8.2
Factory worker	10	3.6
Student	142	50.7
**Occupation types**		
Agriculture	49	17.5
Non-agriculture	231	82.5
**Household monthly income**		
<RM 500 (<US$ 166.7)	183	65.4
>RM 500 (>US$ 166.7)	97	34.6
**Wearing shoes for outdoor activity**		
Yes	192	68.6
No	88	31.4
**Taking bath at least once a day**		
Yes	233	83.2
No	47	16.8
**Changing clothes at least once a day**		
Yes	226	80.7
No	54	19.3
**Keeping animal pets**		
Yes	236	84.3
No	44	15.7

On the socioeconomic surveys, more than half of the populations received a household income of less than US$ 166.7 (RM 500) per month (n = 183, 65.4%). At least half of the population received formal education (6-year of primary education) while 42.9% did not have any formal education.

Upon investigation of their personal habits, a majority of them responded positively on wearing shoes for outdoor activity (68.6%), taking bath and changing clothes at least once a day (83.2%) and keeping animal pets at home (84.3%) (Table 1).

### Antibody prevalence to *O. tsutsugamushi*

Antibody against *O. tsutsugamushi* was detected in 50 (17.9%) participants investigated in this study (Table 2). The antibody prevalence to *O. tsutsugamushi* ranged from 0 to 36.4% in seven subgroups, with the highest prevalence being observed for the Semai Pahang subgroup (36.4%; 95% CI = 1.9–6.7%). This was then followed by the Semelai (31.7%; 95% CI = 17.9%), Temuan (21.1%; 95% CI = 0.9–5.2%), Semai Perak (15.2%; 95% CI = 0.3–3.2%), Temiar (15.1%; 95% CI = 1.4–6.2%), and Orang Kuala subgroups (2.1%; 95% CI = −0.3–1.1). None of the participants from Mah Meri subgroup was positive (0%; 95% CI = 0). Of the age groups analysed in this study, the highest seropositivity was seen among those participants ≥18 years (24.6%), followed by 12–17 years (15.6%) and below 12 years old (6.8%)(Table 2).

**Table 2:** Prevalence of IgG antibodies to *O. tsutsugamushi* among the surveyed population (n = 280).

Aboriginal subgroups	No. seropositive/total population (%)	95% CI
Semelai	13/41(31.7)	17.9
Semai Pahang	12/33(36.4)	1.9–6.7
Temiar	11/73(15.1)	1.4–6.2
Temuan	8/38(21.1)	0.9–4.9
Semai Perak	5/33(15.2)	0.3–3.2
Orang Kuala	1/48(2.1)	−0.3–1.1
Mah Meri	0/14(0.0)	0
**Age groups (years old)**		
Below 12	3/44(6.8)	−0.1–2.3
12–17	19/122(15.6)	3.9–9.8
18 and above	28/114(24.6)	6.5–13.5
**Gender**		
Male	15/112(13.4)	2.8–8.2
Female	35/168(20.8)	8.7–16.4
**Total**	**50/280(17.9)**	**13.4–22.4**

Females (20.8%) had higher seropositivity rate against *O. tsutsugamushi* as compared with males (13.4%). However, there was no significant difference or association between antibody prevalence and gender (P>0.05) (Table 3). Univariate analysis indicated that populations with age above 18 years (OR = 1.15, 95% CI = 1.02–1.30, *P* = 0.015), working (OR = 1.99, 95% CI = 1.01–3.92, *P* = 0.044), working at agriculture area (OR = 1.18, 95% CI = 0.98–1.42, *P* = 0.031), receiving household income less than USD 166.7 (RM500) per month (OR = 2.43, 95% CI = 1.16–5.11, *P* = 0.016) and having close contact with animal pets (OR = 4.06, 95% CI = 1.20–13.76, *P* = 0.016), are significantly associated with exposure to *O. tsutsugamushi*. Multivariate analysis confirmed that participants who are above 18 years old, receiving household income less than USD 166.7 (RM500) per month and having close contact with animal pets were 3.6 times (95% CI = 1.81–7.03, *P*<0.001), 1.3 times (95% CI = 1.14–1.64, *P* = 0.002) and 1.2 times (95% CI = 1.05–1.06, *P* = 0.006), are more likely to have exposure to *O. tsutsugamushi* (Table 3).

**Table 3:** Potential factors associated with exposure to *O. tsutsugamushi* among the surveyed population.

Variables		Scrub typhus seropositive		Crude OR(95% CI)	*p* value	Adjusted OR(95% CI)	*p* value
	N	no	%	1		1	
**Gender**							
Female	168	35	20.8	1.09 (0.98–1.22)	0.111		
Male	112	15	13.4	1			
**Age groups (years old)**							
≥18	114	28	24.6	1.15 (1.02–1.30)	0.015	3.57 (1.81–7.03)	<0.001
<18	166	22	13.3	1		1	
**Level of education**							
No formal education	120	27	22.5	1.12 (0.98–1.24)	0.079		
Formal education	160	23	14.4	1			
**Occupation categories**							
Working	60	16	26.7	1.99 (1.01–3.92)	0.044		
Not working	220	34	15.5	1			
**Occupation**							
Agriculture	49	14	28.6	1.18 (0.98–1.42)	0.031		
Non-agriculture	231	36	15.6	1			
**Household monthly income**							
<RM 500 (<US$ 166.7)	183	40	21.9	2.43 (1.16–5.11)	0.016	11.29 (1.14–1.64)	0.002
>RM 500 (>US$ 166.7)	97	10	10.3	1			
**Wearing shoes for outdoor activity**							
Yes	192	37	19.3	1.38 (0.70–2.74)	0.362		
No	88	13	14.8	1			
**Taking bath at least once a day**							
Yes	233	40	17.2	1.05 (0.90–1.23)	0.502		
No	47	10	21.3	1			
**Changing clothes at least once a day**							
Yes	226	41	18.1	1.11 (0.51–2.45)	0.799		
No	54	9	16.7	1			
**Keeping animal pets**							
Yes	236	47	19.9	4.06 (1.20–13.76)	0.016	1.17 (1.05–1.06)	0.006
No	44	3	6.8	1		1	

N: Number examined; Reference group marked as OR = 1; CI: Confidence integral; Significant association set at *p*<0.05.

## Discussion

Clinical presentation and the history of a patient are important to aid diagnosis of scrub typhus. However, the disease can be difficult to be differentiated from leptospirosis, murine typhus, malaria, dengue and other tropical diseases due to the similarity in their clinical features. The observation of eschar supports the diagnosis of scrub typhus, however; it is not usually present [19]. The mainstay in the diagnosis of scrub-typhus is by serology. However, this approach is usually hampered by the lack of serological assays due to the difficulty in preparing native antigens for *O. tsutsugamushi*. As a result, misdiagnoses and delayed treatment of scrub typhus have been frequently reported in the rural areas; the lack of appropriate laboratory assays has also caused the underestimation of scrub typhus in many parts of the world [1].

This study provides the most recent serologic data for *O. tsutsugamushi* infection in different subgroups of aboriginal population in West Malaysia. The antibody prevalence to *O. tsutsugamushi* varied according to localities. The overall antibody prevalence to *O. tsutsugamushi* (17.9%) in this study was higher than those reported previously for aboriginal settlements in West Malaysia [13] and the indigenous communities in East Malaysia [20]. This study also confirmed the findings of Audy [21] that the epidemiology of scrub typhus is closely related to human occupation and behaviour. Although scrub typhus has been reported from different geographical zones such as seashores, mountainous regions, rainforests, river banks and terrain undergoing secondary vegetation growth, most cases occur through agricultural exposure [22]. Our findings are thus consistent with these earlier observations as higher prevalence rates to *O. tsutsugamushi* are seen with five aboriginal subgroups (i.e., Semelai, Semai Pahang, Temiar, Temuan and Semai Perak subgroups) whose main occupations are associated with agricultural activities, whereas only minimal and zero prevalence was noted in two subgroups (i.e., Orang Kuala and Mah Meri subgroups) whose main occupations were fishing.

Shifting cultivation, which is a normal practice of some aboriginal subgroups, may contribute towards the creation of conducive ecological condition for the transmission of scrub typhus. New cultivated land attracts rodents and animals which carry *O. tsutsugamushi*-infected mites and thus, forms an intensive transmission focus (also called as “mite-island”). When the land is no longer fertile after a period of time, the cultivation is shifted to another piece of land. The outcome of this process is the expansion of the transmission focus for scrub typhus with the simultaneous shifting of infected mites and animal reservoirs to the new cultivated lands. The infectivity of the mite population can be maintained over long periods of time as the infection of adult mites can be passed to their eggs (transovarial transmission) and from the egg to the larva or adult (transstadial transmission) [23], [24].

In this study, multivariate analysis confirmed that participants who were above 18 years old were significantly associated with exposure to *O. tsutsugamushi* (Table 3). The observation of a higher exposure rate in older age group was also noted among febrile patients in rural areas in Malaysia [3]. This phenomenon can be attributed to the increased contact of the participants with an intensive transmission focus or “mite island”, where *O. tsutsugamushi* is found persistently in *Leptotrombidium* mites and animal reservoirs in a specific area. Repeated inoculation of the aboriginal population with *O. tsutsugamushi* in the mite-island may result in long-term persistence of antibody and inapparent, chronic scrub typhus infection [25].

Two Malaysian serosurveys documented comparable or higher prevalence of antibody against *O. tsutsugamushi* in the male participants [3], [26]. Males were generally more active in the outdoor activities like farming, hunting or hiking than the females, and hence, having higher exposure rates to infected mites. However, it was interesting to note that females in this study had higher antibody prevalence to *O. tsutsugamushi* as compared to males (20.8% vs 13.4%). An earlier study by Strickman et al. [27] reported similar observation as they found rural women performing agricultural tasks experienced higher levels of exposure than men. However, no significant difference or association between antibody prevalence to *O. tsutsugamushi* and gender (P>0.05) was noted in this study (Table 3).

The endemicity of scrub typhus in the Asia Pacific region has been correlated with people in rural areas who are exposed to environmental factors such as bushes, piles of wood, domestic animals and rodents [28]. In this study, majority of the participants receiving household income less than USD 166.7 (RM500) per month were significantly associated with exposure to *O. tsustsugamushi* (Table 3). The poverty, lack of awareness and proper protective measures for scrub typhus disease could have contributed to the high exposure of the aboriginal communities to *O. tsustsugamushi*-infected mites.

Institution of good personal hygiene may reduce risk of acquiring scrub typhus infection. As mites require 36–72 hours to attach to the skin of the host, hence, thorough scrubbing and washing of the body after exposure may decrease the risk of mite bites and, thus, the risk of scrub typhus [29]. Sharma et al. [28] reported that it is less likely to acquire scrub typhus when one bath after work and change clothes to sleep. However we did not observe significant difference in the antibody prevalence to *O. tsutsugamushi* for individuals who took bath and changed clothes at least once a day (Table 3). Similarly, the practice of wearing shoes for outdoor activity (as responded positively by 192 participants in this study) did not have significant effect on the exposure to *O. tsutsugamushi* in this study. Instead, wearing gumboots has been associated with a lower risk of acquiring scrub typhus in a recent survey in India [28].

The multivariate analysis in this study confirms that participants keeping animal pets are significantly associated with exposure to *O. tsutsugamushi* (Table 3). Peridomestic animals such as dogs and cats can serve as transport hosts as they harbour infected mites and may lead to exposure of aboriginal population to scrub typhus [30], [31]. In addition, the leftover food for domestic animals attracts rodents and households frequented by rodents could be more affected by scrub typhus [32], [33], whereas clean living-environment and control of rodents decreased the incidence of scrub typhus significantly among troops in China [34].

In conclusion, this study presents evidence that scrub typhus remains an important disease amongst various aboriginal subgroups and confirms that previous findings still apply after many years, highlighting neglected issues related to scrub typhus, a treatable and preventable disease. The environment, socioeconomic, and behavioural risk factors which have a significant relationship to the risk of exposure to scrub typhus have been identified. As it was not feasible to study the entire population of aboriginal population, a sample of the population consisting of 280 individuals from 7 aboriginal subgroups was included in this study. The small numbers of participants is considered one of the limitations of the study as the resulting random sampling error might give some implications on the statistical analysis and conclusion drawn [35]. To minimize such error, continued surveillance for scrub typhus in the aboriginal community is necessary and recommended. The data obtained would be beneficial to the health authority in designing better prevention and control strategies for scrub typhus in the aboriginal population in West Malaysia. Awareness about the disease and education on the preventive measures such as clearing of bushes, use of protective clothing, keeping animals away and controlling rodents are important to reduce the risk of acquiring scrub typhus in the population studied.

## Supporting Information

Checklist S1 STROBE, checklist.
(DOC)
